# Compressive Fracture Behavior of Zirconia/Resin Composites Prepared by Fused Deposition Modeling Combined with Vacuum Infiltration

**DOI:** 10.3390/ma17091989

**Published:** 2024-04-25

**Authors:** Xiaole Yang, Jinyu Guo, Yuanbing Li, Xianfeng Yang

**Affiliations:** 1 The State Key Laboratory of Refractories and Metallurgy, Wuhan University of Science & Technology, Wuhan 430080, China; yangxl_0720@163.com; 2 College of Materials Science and Engineering, Changsha University of Science & Technology, Changsha 410014, China; guojinyu0519yxl@163.com; 3 National-Provincial Joint Engineering Research Center of High Temperature Materials and Lining Technology, Wuhan University of Science and Technology, Wuhan 430081, China

**Keywords:** fused deposition modeling, ZrO_2_/resin composite, porous structure, compressive strength

## Abstract

Although bioceramic materials exhibit good biocompatibilities and bone conductivities, their high brittleness and low toughness properties limit their applications. Zirconia (ZrO_2_)/resin composites with idealized structures and properties were prepared by fused deposition modeling (FDM) combined with a vacuum infiltration process. The porous structure was prepared using the FDM three-dimensional printing technology, with granular zirconia as the raw material, and the relationship between the pore shape, pore size, and deformation was discussed. The results showed that square pores were more suitable than honeycomb pores for printing small pore sizes, and the resolution was high. Scanning electron microscopy observations showed that the superposition of multiple printing paths promoted the emergence of hole defects. The effects of the resin and the pore shape on the compressive strengths of the composites were studied. It was found that the compressive strengths of the honeycomb pore ZrO_2_/resin composites and porous ceramics were superior to those of the square pore samples. The introduction of the resin had a significant effect on the compressive strengths of the composites. The compressive strength increased in the direction perpendicular to the pores, while it decreased in the direction parallel to the pores.

## 1. Introduction

Bioceramic materials have attracted increasing attention because of their good biocompatibilities and bone conductivities. However, their high brittleness and low toughness properties limit their applications [1]. Inspired by natural materials, such as bone and nacre from seashells, the ceramic/polymer composite strategy is considered an effective means to improve the comprehensive performances of bioceramics [2,3,4,5]. Ceramic/polymer composites are expected to possess the properties of both ceramics and polymers to overcome the typical brittle behavior of ceramics and increase their stiffness attributes [6,7,8]. Ceramic/polymer composites are widely used in biomedical fields such as bone tissue engineering and dentistry. In bone tissue engineering, traditional fabrication techniques, including electrospinning [9,10,11], gas foaming [12,13], solvent casting-particulate leaching [14,15], and phase separation [16,17,18] are widely used to fabricate the scaffolds [19]. In dentistry, polymer/ceramic composites are fabricated by dispersing ceramic particles in a polymer matrix, or by infiltrating polymers into a pre-sintered glass–ceramic scaffold to generate a polymer-infiltrated ceramic network (PICN) [20,21,22,23,24,25]. These traditional preparation methods have the drawback that the ceramic particles are difficult to be uniformly dispersed in the polymer matrix, and the pore structure of the ceramic matrix cannot be accurately controlled [26,27]. Furthermore, to design and prepare these composites, computer-aided design and computer-aided manufacturing (CAD-CAM) systems must be employed, which are time-consuming and expensive.

Three-dimensional (3D) printing is a technical realization form of additive manufacturing (AM); it refers to a series of technologies used for the manufacture of solid components in a layer-by-layer approach based on the computer-aided design (CAD) model [28,29,30]. This enables the preparation of 3D components exhibiting near net and complex geometrical shapes. It is known to reduce preparation costs and facilitate production efficiencies, and consequently, it has been widely used in the aerospace, automotive, energy, and biomedical fields, among others [31,32,33,34]. When applied to porous ceramics, 3D printing can accurately control the pore shape, porosity, and connectivity of the pores, characteristics that are difficult to achieve using traditional fabrication methods. At present, there are few reports on the preparation of ceramic/resin composites using the 3D printing technology. In particular, systematic evaluations are lacking with regard to the reliability of the porous ceramic matrix and the compressive fracture behaviors of the ceramic/resin composites. According to the type of raw material employed, 3D printing technologies can be categorized into slurry-, powder-, and bulk solid-based methods [35]. Among the various 3D printing technologies reported to date, fused deposition modeling (FDM) has attracted considerable attention because of its low equipment costs, facile operation, and low raw material costs [36,37,38,39]. The feedstocks employed in FDM are typically composed of ceramic powders and organic binders, which are mixed under a strong shear force to eliminate powder agglomeration and maintain a stable dispersion during storage and printing. As a result, precipitation and segregation are eliminated from this process. Traditional FDM printers typically use flexible and fixed-diameter filaments as raw materials; however, flexible ceramic filaments are difficult to prepare, and ceramic filaments with high solid loadings can break easily.

In our preliminary work [26], an FDM system based on screw extrusion was developed for feeding granular feedstocks with high solid loadings. The printed raw materials were composed of a ceramic powder and a thermoplastic organic carrier, which were mixed together under the action of a strong shear force to prepare the granular feedstock. The feedstock was extruded using a screw extruder at high temperatures to obtain a compact and uniform body with a high solid loading.

Thus, in the current study, ZrO_2_/resin composites were prepared using FDM 3D printing technology combined with a vacuum infiltration process, which provides a new strategy for improving the high brittleness and low toughness of bioceramic materials. The printing performance of the zirconia feedstock and the internal defects of the pore wall were studied, and the compressive mechanical behavior and crack characteristics of the resulting ZrO_2_/resin composites were evaluated. This work is expected to provide some reference for the research and application of ceramic/resin composites prepared by 3D printing.

## 2. Materials and Methods

### 2.1. Materials

Commercially available zirconia powder (Hebei Hengbo New Materials Technology Co., Ltd., Handan, China) with an average particle size of 0.27 μm and a specific surface area of 8.7 m^2^/g was used. The organic binders included polyethylene (PE, USI Corporation, Taiwan, China), ethylene–vinyl acetate copolymer (EVA, Organic Chemical Plant of Beijing Oriental Petrochemical Co., Ltd., Beijing, China), stearic acid (SA, Hebei Delun Chemical Technology Co., Ltd., Shijiazhuang, China), and paraffin wax (PW, Sinopec Jingmen Petrochemical Complex, Jingmen, China). The experimental resin was prepared using bisphenol A glycerolate dimethacrylate (Shanghai Aladdin Biochemical Technology Co., Ltd., Shanghai, China), triethylene glycol dimethacrylate (TEGDMA, West Asia Chemical Technology (Shandong) Co., Ltd., Linyi, China), and benzoyl peroxide (BPO, Shanghai Aladdin Biochemical Technology Co., Ltd., Shanghai, China).

### 2.2. Preparation of the Porous Zirconia Ceramics

The ceramic powder and organic binder were mixed with a double roller mixer (SK-160, Dongguan Jiutong Machinery Manufacturing Co., Ltd., Dongguan, China) at 135 °C for 40 min to prepare the zirconia feedstocks (solid loading of 87 wt.%). The formula of zirconia feedstock was shown in Table 1. Subsequently these components were allowed to cool to room temperature (24–26 °C), and broken down into particles with diameters of <3 mm.

The 3D model was designed using the CAD approach, and the model was imported into the slicing software (UPRISE3D-3.48.108). The printing parameters were set and sliced to generate the G code, which was then imported to a fused deposition molding printer (UP-R200; Shenzhen Uprise 3D Technology Co., Ltd., Shenzhen, China) for printing. The zirconia feedstock was fed into the barrel using a screw, heated to a molten state at 140 °C, and extruded from the nozzle. The porous zirconia green body was prepared in a layer-by-layer approach and then immersed in kerosene at 40 °C for 24 h to ensure solvent de-binding. After drying in an oven at 60 °C for 24 h, the dried green body was heated to 600 °C in a thermal de-binding furnace (RPJ-18-6, Yixing Wanlong Electric Furnace Co., Ltd., Yixing, China) to promote de-binding. Finally, sintering was carried out in a furnace (JXL1600D, Shanghai Jiugong Electric Co., Ltd., Shanghai, China) at 1560 °C for 2 h to obtain the porous zirconia ceramic.

### 2.3. Preparation of the ZrO_2_/Resin Composites

Initially, Bis-GMA and TEGDMA (6:4 mass ratio) were mixed via ultrasonic oscillation for 2 h. After this time, 0.5 wt.% BPO was added, and the mixture was stirred magnetically for 2 h to ensure complete dissolution. The resin was then slowly stirred for a further 1 h under a vacuum of −0.1 MPa to eliminate any air bubbles from inside the resin.

**Figure 1:**
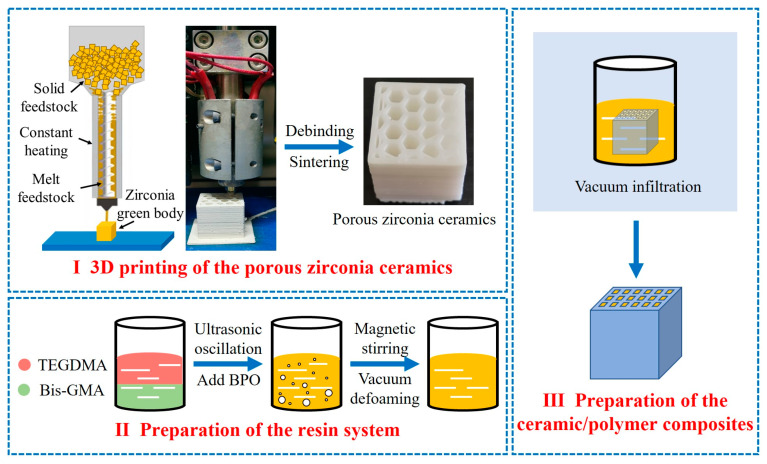
Outline of the process used to prepare the ZrO_2_/resin composites.

The porous zirconia ceramics were immersed in the resin system and placed in a vacuum drying oven (DZF-6050, Shanghai Yiheng Scientific Instrument Co., Ltd., Shanghai, China), under a vacuum of −0.1 MPa for 1 h. Finally, the ceramic/resin composites were obtained by polymerization at 100 °C under a −0.1 MPa vacuum for 12 h. The preparation process is outlined in Figure 1.

### 2.4. Characterization of the Microstructures and Mechanical Properties

The microstructures of the samples were observed using scanning electron microscopy (SEM; JSM-7900F, JEOL, Tokyo, Japan). The densities of the zirconia ceramics were measured using the Archimedes’ method [40,41]. The universal material testing machine (PT-1176, Baoda Instrument Co., Ltd., Xian, China) was used to test the three-point flexural strength. Each group had 35 samples (3 mm × 4 mm × 40 mm), and the results were averaged. The span is 35 mm, and the loading rate of the indenter is 0.2 mm/s. The compressive strengths of the samples (20 mm × 20 mm × 20 mm) were measured using a universal testing machine (WAW-200, Shanghai Bairoe Test Instrument Co., Ltd., Shanghai, China). Three specimens of each sample type were selected for the static compression tests, which were performed at a loading rate of 0.013 mm/s, using displacement control.

## 3. Results and Discussion

### 3.1. Printing of the Porous Structure and Evaluation of the Pore Wall Defects

The printability of the feedstock is a key factor in the FDM process, especially for complex parts, such as lattice structures that do not bear a support. The printability of the zirconia feedstock was tested in our previous study [42]. It was found that the feedstock exhibited a shear thinning property in the molten state; the unsupported tilt angle of the printed sample reached 150°, and the unsupported printing wire span reached 5.5 mm. However, when printing porous structures, the feedstock must meet special printability requirements. Thus, in the current study, the printability of the feedstock was evaluated with regard to three aspects, namely the unsupported porous structure, the minimum printable pore size, and the internal defects of the pore wall.

**Figure 2:**
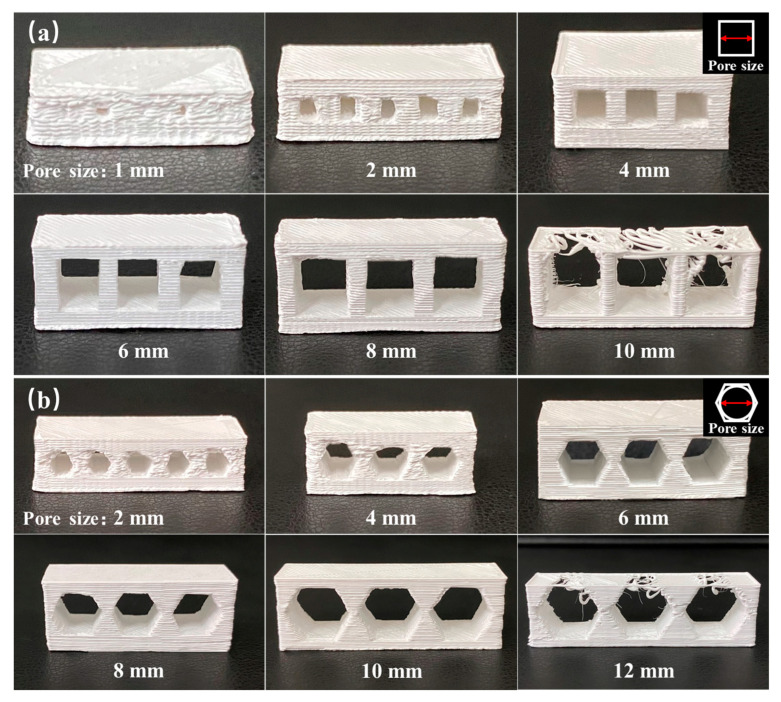
Unsupported porous structures with different pore shapes: (**a**) square pores and (**b**) honeycomb pores.

Using a nozzle diameter of 0.3 mm, unsupported porous structures with different pore shapes were printed using the FDM approach, as shown in Figure 2. As shown in Figure 2a, which presents the printing results for the square pore structure, at a pore size of <4 mm, the square shape was deformed, and pores with a size of 1 mm became blocked. This was attributed to the low precision of FDM technology. More specifically, the wire extruded from the nozzle undergoes compression during filling, which is influenced by parameters such as the extrusion flow, nozzle diameter, and layer thickness. For example, if the extrusion output is too small, pore defects may appear inside the body, and if the extrusion output is too large, material overflow will occur on the side steps, leading to a decrease in the printing accuracy, which is especially noticeable when the difference between the nozzle diameter and the aperture is small. As the pore size increases, this effect gradually decreases. Indeed, a square pore with an aperture of 4–8 mm possessed a clear outline and a complete shape. Upon increasing the square pore size to 10 mm, the extruded wire of the first layer deformed and broke under the action of gravity owing to the large span, which could not provide sufficient support for the subsequent printing layer, and resulted in printing failure. Figure 2b shows an unsupported honeycomb pore structure, wherein the size of the honeycomb pore correlates with the diameter of the inscribed circle. When the pore size was <6 mm, the honeycomb pore shape was deformed, and the accuracy was poor. In contrast, for pore sizes of 6–10 mm, the honeycomb pore shape was clear, and a uniform side-step thickness was achieved. For a larger pore size of 12 mm, the span of the pore wall reached its limit, and printing failed. Compared with square pores, honeycomb pores contain a greater number of edges, which appears to result in a superior bearing capacity but higher printing accuracy requirements. The obtained results demonstrated that the zirconia feedstock used in these experiments exhibited a good rigidity, thereby permitting the preparation of an unsupported porous structure with a pore size of 4–10 mm.

**Figure 3:**
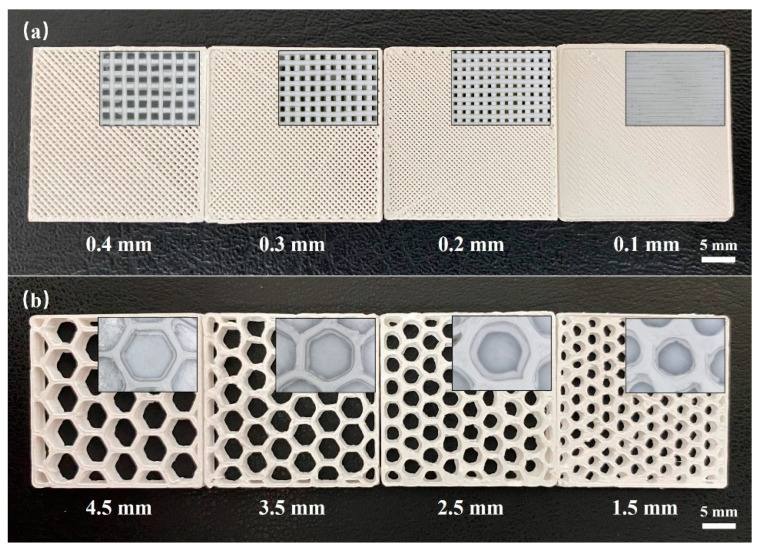
Minimum printable pore sizes for the different pore shapes: (**a**) square pores and (**b**) honeycomb pores.

In practical applications, the pore shapes and sizes significantly affect the mechanical and functional properties of porous materials. Considering that a high printing accuracy is required for printing small pores, the minimum printable pore sizes were evaluated for different pore shapes, as presented in Figure 3. Using a nozzle diameter of 0.3 mm, a square pore structure with a pore size of ≥0.2 mm was successfully printed (Figure 3a). However, for a pore size of 0.1 mm, the printing of the pore structure was unsuccessful. In the case of the honeycomb pore shape, pores measuring ≥ 3.5 mm were successfully printed (Figure 3b), while the honeycomb shape was lost at pore sizes ≤ 2.5 mm, leading to pore deformation. Overall, it was found that to prepare small pores measuring 0.1–3.5 mm in diameter, the printing of square pores was most suitable.

**Figure 4:**
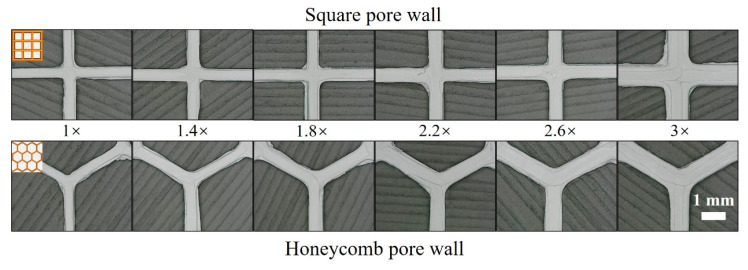
Pore walls of the square and honeycomb porous structures with different thicknesses (1, 1.4, 1.8, 2.2, 2.6, and 3 times the nozzle diameter).

The strength of a porous structure depends on the strength of the pore walls. During the printing process, the interlayer binding and printing path binding are known to significantly influence the strength of the pore walls. In particular, for the preparation of thin pore walls, the printing path is mainly determined by the nozzle diameter, which defines the number and arrangement of the printing paths. In the current experiments, a nozzle with a diameter of 0.3 mm was used to print the porous structures, and the pore wall thickness was set to 1, 1.4, 1.8, 2.2, 2.6, or 3 times the nozzle diameter to explore the influence of the nozzle diameter on the internal defects of the pore walls. Figure 4 shows the pore walls of the square and honeycomb porous structures printed with different thicknesses. It can be seen from the printing layers of these pore walls that the number and arrangement of the printing paths are significantly different. These differences affect the internal conditions of the pore walls, such as the formation of pore defects.

**Figure 5:**
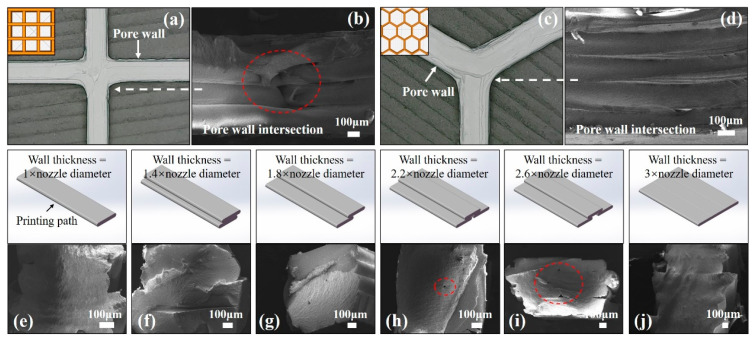
Influence of the nozzle diameter on the internal defects of the pore wall. (**a**) The square pore wall. (**b**) Intersection of the square pore wall. (**c**) The honeycomb pore wall. (**d**) Intersection of the honeycomb pore wall. (**e**–**j**) The cross section of the pore wall.

SEM images of the surfaces and cross-sections of the pore walls are shown in Figure 5. In the case of the square pore wall (Figure 5a), holes can be clearly observed at the intersection (Figure 5b); however, these holes exist only on the surface and do not extend to the interior. In contrast, no holes were formed at the intersection of the honeycomb pore walls (Figure 5c,d) due to the larger inner angle of the honeycomb shape (i.e., 120° c.f., and 90° for the square). This can reduce accumulation of the extruded material and prevent the formation of a blind hole on the surface. For the samples prepared using thicknesses of 2.2 and 2.6 times the nozzle diameter, it can be seen from the SEM images that their pore walls were composed of three printing paths that were superimposed with one another. According to the microscopic morphologies of the pore wall sections (Figure 5e–j), these three printing paths were superimposed at multiple places within the same layer, and it was observed that the bonding of the printing wires was not sufficiently close to generate holes. These defects evolved into pores inside the sintered body, which affected the final strength of the pore wall. Therefore, when printing a thin pore wall, the nozzle diameter should be carefully selected to avoid the superposition of multiple printing paths.

### 3.2. ZrO_2_/Resin Composites and Compressive Mechanical Behaviors

**Figure 6:**
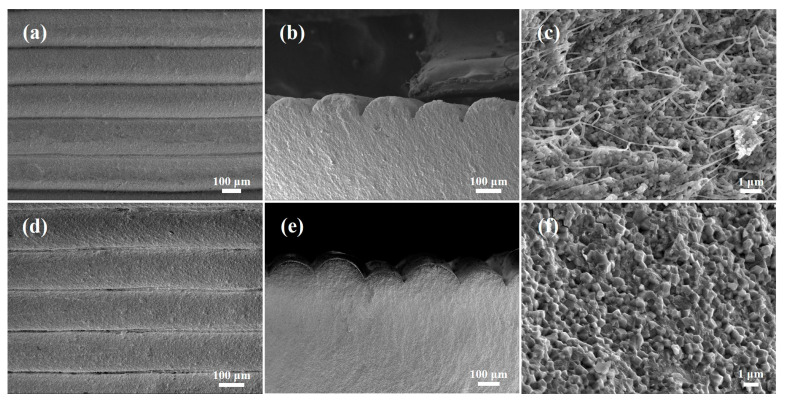
SEM images of the samples prepared by FDM technology. (**a**) Green body surface along the *z*-axis direction. (**b**) Cross-section close to the surface of the green body. (**c**) Cross-section of the green body. (**d**) Sintered body surface along the *z*-axis direction. (**e**) Cross-section close to the surface of the sintered body. (**f**) Cross-section of the sintered body.

The surfaces of samples prepared using the FDM technology are known to exhibit step textures, and if the gap or interface between the surface steps extends to the inner part of the green body, the reliability of the material is reduced. Figure 6a shows the surface of the prepared green body stacked along the *z*-axis, wherein each layer on the surface is clearly uniform and continuous, without any deformation or cracking. In addition, it is known that the gap size is directly related to the nozzle size, layer thickness, and nozzle flow rate. As shown in Figure 6b, the gaps or interfaces on the surface do not extend to the inner part of the green body, and the interior near the surface is dense and without cracks. Figure 6d,e show the SEM images of the surface along the *z*-axis direction and the cross-section of the pore wall of the sintered body, respectively. It can be seen that the microstructural characteristics are consistent with those before sintering, without deformation or cracking. As presented in Figure 6c, thread-like organic binders are evenly distributed inside the green body, which ensures the stiffness of the extrusion wire and the strength of the green body, in addition to providing sufficient support to resist the internal stress caused by debinding and sintering. Notably, the zirconia ceramics sintered at 1560 °C were found to possess a uniform crystal grain size and a dense structure, as shown in Figure 6f; the relative density of this specimen was determined to be 6.0 g/cm^3^, while its flexural strength was 693 ± 45 MPa.

**Figure 7:**
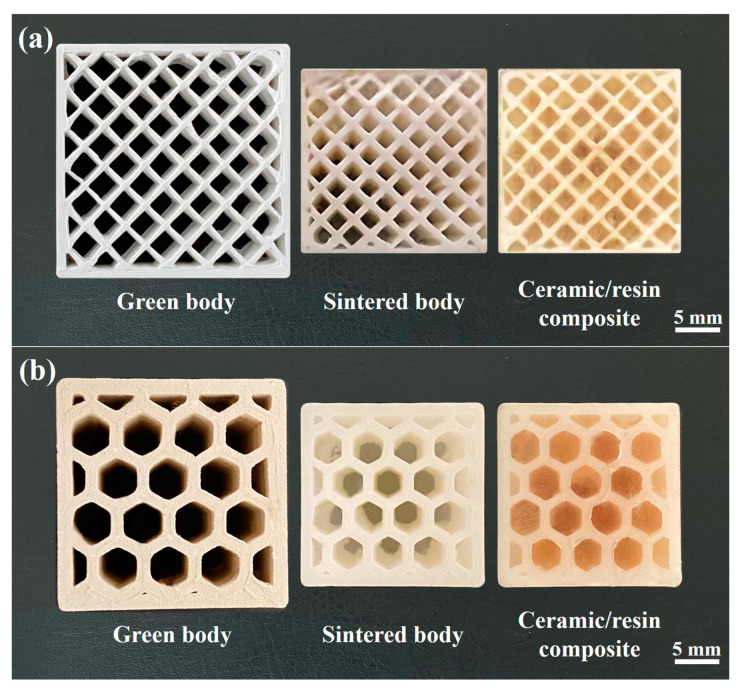
Porous zirconia samples: green body, sintered body, and ceramic/resin composite. (**a**) The square pore structures and (**b**) the honeycomb pore structures.

Figure 7 shows photographic images of the porous zirconia samples bearing square and honeycomb pores, including the green bodies, sintered bodies, and ceramic/resin composites. It can be seen that, after sintering, the square and honeycomb pore shapes remained intact, and the pore walls did not deform or break; the linear shrinkage was 20.3%. In addition, the porosity of the ceramic was determined to be 35%, wherein the resin fully filled all pores without bubble formation.

**Figure 8:**
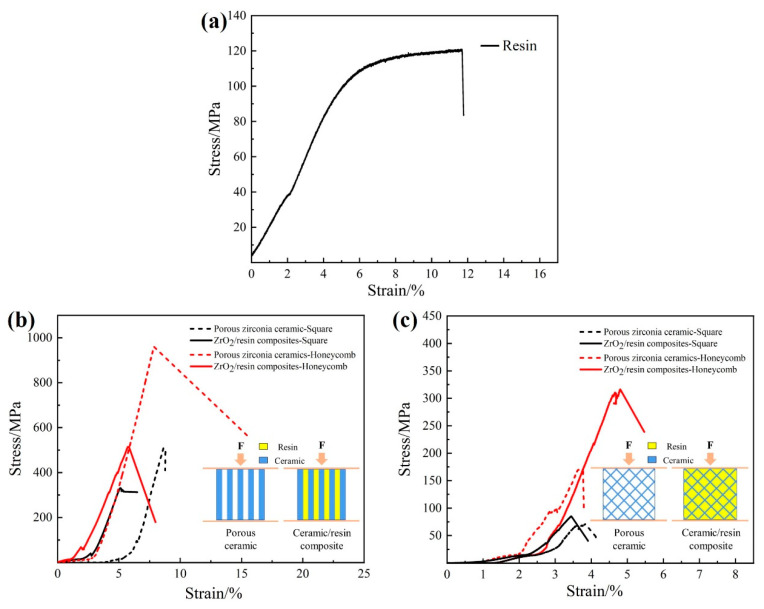
Stress–strain curves of the porous zirconia ceramics and the ZrO_2_/resin composites. (**a**) Compressive strength of resin, (**b**) compression parallel to the pore direction, and (**c**) compression perpendicular to the pore direction.

The effects of the pore shape on the compressive mechanical behaviors of the porous ceramics and the ceramic/resin composites were also investigated. Compression tests were carried out in directions both parallel and perpendicular to the pores. As shown in Figure 8b, when the compressive stress was parallel to the pore direction, the compressive strength of the honeycomb-shaped sample was higher than that of the square-shaped sample, indicating that the honeycomb shape provided greater support to the vertical pore walls than the square shape. However, the compressive strength decreased significantly after the addition of the resin due to the low resin strength (Figure 8a), which led to fracture after compression. Subsequently, the displacement and stress generated by the fracture acted on the pore wall, causing the pore wall to break. When the compressive stress was perpendicular to the pore direction, the compressive strength of the honeycomb-shaped sample was again superior to that of the square-shaped sample, as shown in Figure 8c. This was attributed to the more stable honeycomb shape (a regular hexagon) compared to the square shape. The former exhibits a high energy absorption capacity, which can effectively disperse pressure and prevent crack propagation [43,44]. In addition, when the pressure was perpendicular to the pore direction, the stress was primarily concentrated at the intersection of the pore walls, which quickly reached the strength limit and was prone to fracture. Consequently, the compressive strength perpendicular to the pore direction was lower than that parallel to the pore direction. Furthermore, in contrast to the case of the compressive strength parallel to the pore direction, resin penetration was found to increase the compressive strength perpendicular to the pore direction. This was mainly attributed to the fact that the resin bears part of the stress and plays a role in transmitting it, thereby reducing the stress concentration at the intersection of the hole wall. As a result, the compressive strength is significantly improved perpendicular to the hole direction.

In the above experiments, irrespective of the pressure direction, the compression of the ZrO_2_ ceramic/resin composites led to brittle fracture. Due to the large pore sizes of the porous ceramics, the resin was relatively concentrated and was not well dispersed within the ceramic skeleton. As a result, resin toughening of the porous ceramics was not obvious. Unfortunately, this was not the desired result, and so follow-up work will focus on the development of resin-toughened porous ceramics.

**Figure 9:**
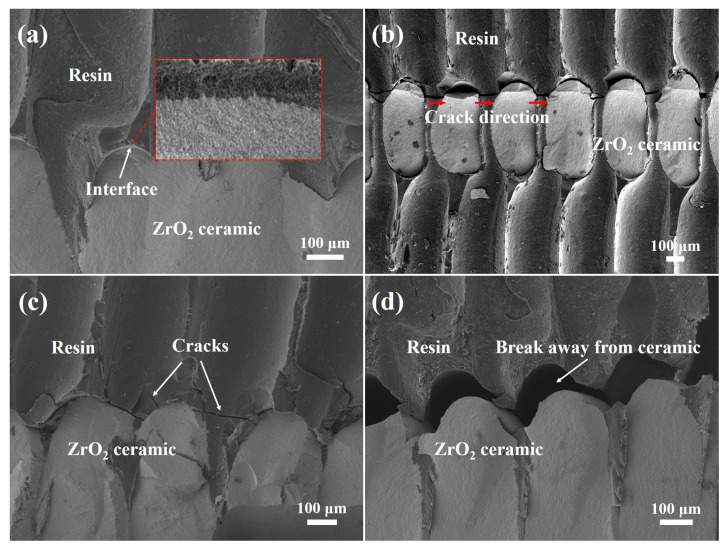
Interface fracture behavior of the ZrO_2_ ceramic/resin composite: (**a**) interface bonding of the ceramic/resin, (**b**) identification of the crack direction, (**c**) resin fracture, and (**d**) detachment of the resin from the ceramic.

Finally, the interface bonding between the ceramic and the resin was evaluated. As shown in Figure 9a, prior to the destruction of the ZrO_2_ ceramic/resin composite by pressure, the zirconia ceramic was tightly bonded to the resin without any cracks or holes. Subsequently, the resin broke under pressure, and cracks were generated along the ceramic pore wall, as shown in Figure 9b. Two main failure modes were identified for the ZrO_2_ ceramic/resin composites under pressure. More specifically, in the first instance, the resin breaks between the steps of the ceramic surfaces (see Figure 9c), whilst in the second instance, fracture occurs at the interface between the ceramic and the resin, and the resin breaks away from the ceramic under pressure (Figure 9d). It was therefore deduced that the fracture behaviors of zirconia ceramic/resin composites mainly depend on the strength of the resin and the interfacial bonding strength between the ceramic and the resin.

## 4. Conclusions

Zirconia (ZrO_2_)/resin composites with resin uniformly distributed in the ceramic matrix were prepared by fused deposition modeling (FDM) combined with a vacuum infiltration process. The zirconia feedstock was found to exhibit a good rigidity, thereby rendering it suitable for the preparation of unsupported porous structures with pore sizes of 4–10 mm. The square pores were more suitable for printing small porous structures, and the minimum printable pore size was determined to be 0.2 mm. When printing the thin hole wall, it was essential to carefully select the nozzle diameter to avoid the formation of internal hole defects, which are caused by the superposition of multiple printing paths. After sintering at 1560 °C, the FDM three-dimensionally printed zirconia ceramic exhibited a density of 6.0 g/cm^3^ and a flexural strength of 693 ± 45 MPa. It was also found that the honeycomb pores provided a greater amount of support for the porous structure. More specifically, the compressive strengths of the honeycomb ZrO_2_/resin composites and porous ceramics were superior to those of the samples based on square pores. Furthermore, the compressive strength was reduced in the presence of the resin when compression was parallel to the pore direction. The compressive strength of the ZrO_2_/resin composites containing honeycombed pores was 514 MPa (compression parallel to the pore direction). Notably, the compressive strength of the ZrO_2_/resin composites containing honeycomb pores was 310 MPa (compression perpendicular to the pore direction). The results show that the resin significantly improves the compressive strength of the composite material when compression is perpendicular to the pore direction.

## Figures and Tables

**Table 1:** The formula of zirconia feedstock for FDM 3D printing.

ZrO_2_ Powder	PW	SA	EVA	PE
87 wt.%	7.5 wt.%	0.7 wt.%	2.2 wt.%	2.6 wt.%

## Data Availability

Data are contained within the article.
